# Nurse Coaching in Oncology Care to Reduce Stress: A Systematic Review and Meta-Analysis

**DOI:** 10.3390/healthcare14070840

**Published:** 2026-03-25

**Authors:** Elsa Vitale, Lorenza Maistrello, Karen Avino, Giuseppe Colonna, Ivan Rubbi, Roberto Lupo

**Affiliations:** 1 Healthcare Directions, ASL Bari, 70100 Bari, Italy; 2 IRCCS San Camillo Hospital, 30126 Venice, Italy; lorenza.maistrello@hsancamillo.it; 3 Integrative Nurse Coach Academy, International Nurse Coaching Association, Miami, FL 33132, USA; kavino@inursecoach.com; 4 Gynecologic Oncology Unit, IRCCS Istituto Tumori “Giovanni Paolo II”, 70124 Bari, Italy; g.colonna@oncologico.bari.it; 5 Department of Medical and Surgical Sciences, University of Bologna, 40126 Bologna, Italy; ivan.rubbi2@unibo.it; 6 Department of Surgery, ‘San Giuseppe da Copertino’ Hospital, Local Health Authority (ASL) of Lecce, 73043 Copertino, Italy

**Keywords:** coaching intervention, oncology nursing, stress

## Abstract

**Highlights:**

**What are the main findings?**

- A total of three studies were included, comprising 112 participants. Heterogeneity among studies was very high and statistically significant (*p* < 0.001; τ^2^ = 1.02; I^2^ = 96.2%, with a 95% CI: [85.89; 99.90]); thus, a random-effects model (REML) was applied.
- A small, non-significant reduction in stress levels following the nurse coaching intervention, with an overall SMD of −0.35 (SE = 0.60; 95% CI: [−1.52, 0.82]; *p* = 0.556), was recorded.

**What are the implications of the main findings?**

- Attention to standardization of core coaching components, while preserving flexibility and personalization, will be critical to advancing implementation in routine oncology care.
- Nurse coaching represents a promising adjunct to comprehensive cancer care, with the potential to support psychological well-being, empower patients and caregivers, and address the broader determinants of stress throughout the cancer experience.

**Abstract:**

**Background:** Nurse coaching can reduce stress throughout the complex psychosocial process associated with the cancer journey, which affects numerous spheres, such as neurological, psychological, physical, and emotional ones. The purpose of this paper is to review the literature to assess the extent of stress reduction among cancer care adopting nurse coaching interventions. **Methods:** This systematic review and meta-analysis was registered in PROSPERO with id no. CRD420261290368 and performed according to the Preferred Reporting Items for Systematic Reviews and Meta-analysis (PRISMA) guidelines. The search approach was performed by mixing keywords with Boolean operators like “coaching intervention”, “oncology nursing” and “stress” throughout the CINAHL, Embase, PubMed, Scopus and Web of Science databases. We assessed stress levels alongside the nurse coaching interventions using the National Comprehensive Cancer Network (NCCN) Distress Thermometer. **Results:** A total of three studies were included, comprising 112 participants. Heterogeneity among studies was very high and statistically significant (*p* < 0.001; τ^2^ = 1.02; I^2^ = 96.2%, with a 95% CI: [85.89; 99.90]); thus, a random-effects model (REML) was applied. A small, non-significant reduction in stress levels following the nurse coaching intervention, with an overall SMD of −0.35 (SE = 0.60; 95% CI: [−1.52, 0.82]; *p* = 0.556), was recorded. **Conclusions:** Attention to standardization of core coaching components, while preserving flexibility and personalization, will be critical to advancing implementation in routine oncology care. Overall, nurse coaching represents a promising adjunct to comprehensive cancer care, with the potential to support psychological well-being, empower patients and caregivers, and address the broader determinants of stress throughout the cancer experience.

## 1. Introduction

A diagnosis of cancer often represents a strong psychological event accompanied by fear, anxiety, depression and emotional distress, which significantly and negatively impact both patients’ and caregivers’ ‘quality of life’ (QoL) perception [1]. All these adverse effects may also persist even after treatment conclusion. Emotional distress due to cancer has also been associated with the activation of the neuroendocrine stress pathway which increases stress hormone excretion and emphasizes the immune system dysregulation process [2,3]. Thus, interventions addressed to both psychological and practical sources of stress are pivotal elements for holistic cancer care.

Evidence suggests that nurse coaching is important to ameliorate QoL and reduce stress among cancer patients [4,5]. To date, several nurse coaching interventions have been performed to better face up to stress derived from cancer disease, both in-person and nurse-led coaching, which try to ameliorate QoL perceptions [1,2,3,4]. Nurse coaching can reduce stress through several interrelated mechanisms that highlight the complex psychosocial process associated with cancer [2,3].

Specifically, coaching could be recognized as an empowering cooperation that is a thought-inducing and inventive process, encouraging individuals to improve their individual capacity, with a positive performance goal, that is time-limited and addressed to specific areas of development through action-oriented outcomes [6,7,8].

Nurse coaching may reduce stress in cancer patients or in their caregivers by addressing the complex, multidimensional nature of the disease through a holistic, patient-centered approach. By fostering partnership, emotional validation, and proactive management, nurse coaching helps patients navigate the trauma, loss, and uncertainty associated with a cancer diagnosis [9].

An essential process of nurse coaching is the encouragement, optimization and reinforcement of existing informal and formal support social networks. Identifying inconsistencies in support reduces isolation sensations as an important factor in distress. By normalizing help-seeking behaviors and promoting access to support, nursing coaches ameliorate emotional burden and unpredictability [5].

Nurse coaching emphasizes the development of self-efficacy in clients through nurse education and competency building. Psychoeducational factors shape disease comprehension, treatment perspectives, symptom management, and recovery pathways, thereby reducing uncertainty and fear [10]. Additionally, nurse coaching interventions develop self-compassion by incorporating stress management, meditation, and also secondary related outcomes from stress, such as insomnia management support, providing individuals necessary tools to mitigate emotional responses throughout the cancer journey [11,12].

Patient-centered design and flexibility provide a holistic approach to stress management rather than only management of emotional symptoms [13].

Thus, nurse coaching reduces stress among cancer patients by emphasizing social support, coping skills, and self-efficacy, thereby improving flexibility and accessibility of care. Together, these elements improve psychological well-being and support the importance of the integration of nurse coaching into oncology practice [9].

According to Almutairi, Almutairi, and Alodhialah [14], nurse coaches help lower patient stress in several ways: by building self-confidence and personal empowerment, by supporting better management of symptoms, and by encouraging collaborative decision-making. These coaches guide patients in comprehending their treatment choices and ensuring care plans reflect their individual values, thereby decreasing the overwhelming feeling and worry that often accompany complicated medical decisions. By listening attentively and communicating with compassion, nurses create a safe and accepting space where patients can work through feelings of fear, trauma, and uncertainty. This emotional support helps combat feelings of isolation. Additionally, nurse coaches promote participation in lifestyle and behavior modifications that enhance mental and emotional health [15].

Considering all the evidence highlighted above, we aimed to explore the current scientific literature to assess stress levels in nurse coaching in oncology care, referring both to patients and their caregivers, and to assess if nursing coaching could ameliorate stress perceptions among individuals involved in the cancer journey. Thus, the present systematic review and meta-analysis aimed to assess stress reduction levels among subjects who experienced or assisted cancer treatment adopting nurse coaching interventions.

## 2. Materials and Methods

### 2.1. Search Approach

This systematic review and meta-analysis was registered in PROSPERO with id no. CRD420261290368 according to the Preferred Reporting Items for Systematic Reviews and Meta-analysis (PRISMA) guidelines [16].

The search was performed by mixing keywords with Boolean operators like “coaching intervention”, “oncology nursing” and “stress”, whilst also including in the “Population–Intervention–Outcome” (PIO) arrangement (Table 1), throughout the CINAHL, Embase, PubMed, Scopus and Web of Science databases. We assessed stress levels among subjects who experienced or assisted a cancer treatment adopting nurse coaching interventions using the National Comprehensive Cancer Network (NCCN) Distress Thermometer. The NCCN Distress Thermometer assessed multidimensional stress dimensions that make it difficult to cope with cancer diagnosis, symptoms, or treatment [17].

### 2.2. Inclusion and Exclusion Criteria

The review included all interventional studies recording nurse coaching effects in stress reduction among adults who experienced or cared for cancer (age ≥ 18 years).

Specifically, we included studies assessing stress levels thanks to the NCCN Distress Thermometer before and after any nursing coaching interventions, both among cancer patients and caregivers. Additionally, no time limit was introduced in our search, as well as no language restrictions. On the other hand, we excluded studies which did not assess stress-related variations in nursing coaching interventions in oncology care. Additionally, we excluded all posters, abstracts, and letters to the editor with any reporting data.

### 2.3. Data Extraction

Initially, records were recognized through a systematic database search and uploaded manuscripts to remove duplicates. Then, two independent reviewers (E.V. and L.M.) assessed the title and abstract of the identified studies for inclusion, and unsuitable reports were removed. Subsequently, the articles were uploaded, and the full texts were assessed more closely for eligibility. Disagreements in the inclusion process were solved thanks to arbitration from another reviewer (K.A.). Data collection included author, year of publication, participants (cancer typology), coaching interventions, specifically the intervention in nurse coaching in setting, intent and description.

**Figure 1:**
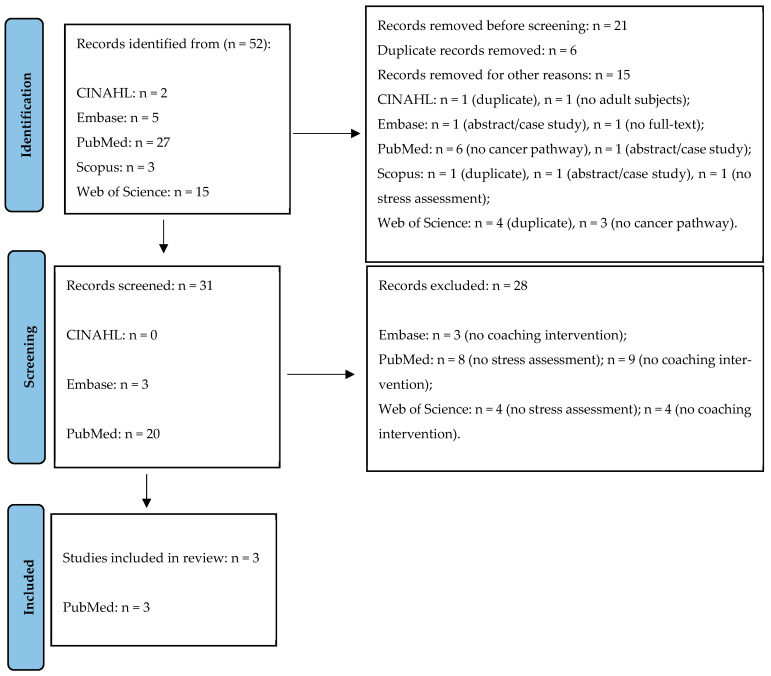
PRISMA flow chart.

Figure 1 explains all search processes according to the PRISMA statement.

### 2.4. Risk of Bias Evaluation

**Figure 2:**
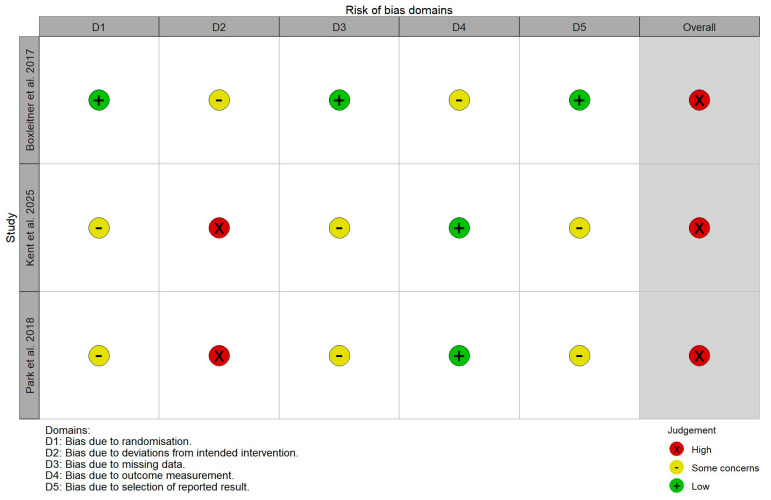
Risk of bias evaluation [19,20,21].

Risk of bias was assessed thanks to the ROB 2 tool [18], consisting of a total of five sub dimensions, like biases arising from the randomization process, deviations from the intended interventions, the lack of outcome data, outcome measurement and the selection of the reported outcome (Figure 2).

### 2.5. Quality Assessment

The quality assessment of each study included in the present systematic review and meta-analysis was elaborated considering their designs and related levels of evidence according to the evidence-based nursing (EBN) approach [22], ranging from level I, indicating the strongest quality, to level VII, suggesting the weakest quality, as follows:

- Level I: Evidence from scoping reviews or meta-analyses of randomized controlled trials;
- Level II: Evidence from well-designed randomized control trials;
- Level III: Evidence from well-designed control trials that are not randomized;
- Level IV: Evidence from case-control or cohort studies;
- Level V: Evidence from scoping reviews of descriptive or qualitative studies;
- Level VI: Evidence from a single descriptive or qualitative study;
- Level VII: Evidence from expert opinions.

Quality assessments were performed by three independent reviewers (G.C., R.L., and E.V.), and in case of disagreement, another reviewer was consulted (I.R.). Finally, only articles belonging to Levels I–III were included in the scoping review. Additionally, scoping reviews and meta-analyses were manually searched in their related references, and further quality assessments were performed.

### 2.6. Data Synthesis

Data extracted from the final selected studies were presented using a narrative approach. Statistical analysis was performed using the R environment (version 4.5.2), with a significance threshold set at *p* < 0.05. The primary objective of this meta-analysis was to assess whether stress levels change following a nurse coaching intervention by comparing baseline and follow-up measurements. To address this objective, effect sizes were calculated using the Standardized Mean Change with raw score standardization (SMCC), which accounts for the correlation between pre- and post-intervention measurements within the same participants [23]. For each eligible study, the SMCC was computed as the standardized difference between post-intervention and baseline mean stress levels, with an assumed within-subject correlation of r = 0.5 due to the absence of reported values. Sensitivity analyses were performed using alternative correlations (r = 0.3 and r = 0.7) to assess the robustness of the results [23,24]. Effect sizes were pooled using a random-effects model with REML estimation. Statistical heterogeneities were assessed using the χ^2^-based Q test, with a *p*-value < 0.05 indicating statistically significant heterogeneity. In addition, heterogeneity was quantified using the I^2^ inconsistency index, with values of <25%, 25–50%, 50–75%, and >75% interpreted as low, moderate, high, and very high heterogeneity, respectively [25,26]. Given the anticipated small number of included studies, I^2^ estimates were interpreted with caution and were reported together with their 95% confidence intervals. Pooled effect sizes were estimated using a random-effects model, implemented through restricted maximum likelihood (REML), as heterogeneity across studies was expected due to differences in study populations, intervention characteristics, and stress measurement instruments. Forest plots were generated to visually summarize individual study effects and the overall pooled estimate [27,28].

## 3. Results

### 3.1. Selected Studies

A total of three studies were included in the present systematic review and meta-analysis. Table 2 displays all data referring to coaching interventions in oncology nursing.

Evidence from the Boxleitner et al. [19] study suggested the efficacy of meditation support during radiation therapy and identified key coaching values, such as flexibility and patient autonomy. This approach enabled participants to reflect on the prioritization of patient choice and adaptability. In this way, patients recorded lower anxiety and distress levels, adopting few intensive resources [29,30]. These benefits underscored the effectiveness of nurse coaching approaches, which use guidance and skill development rather than continuous direct supervision.

The enCompass Carolina program proposed an achievable, suitable, coaching-based supportive intervention for caregivers of rural cancer patients. Caregivers rated this coaching intervention as very satisfactory, since this approach was developed on a caregiver-centered coaching model that specifically addressed the needs of rural populations, including both informal and formal support systems, specifically institutionalized or not, to improve support-seeking skills [22,23]. In this coaching approach, the use of an easy digital guiding tool, which helped caregivers recognize present social support, identified the promising role of family and friends, and focused on unmet support requirements through linkage to further resources. This organized coaching approach provided caregiver meditation, problem-solving, and empowerment.

Similarly, individualized psychoeducational interventions for breast cancer patients were addressed in Park et al.’s study [21] thanks to coaching-based approaches by addressing patient-recognized origins of distress in cancer. Specific education programs addressing emotional well-being and quality-of-life-related issues were developed to emphasize understanding of cancer and its treatment-related symptoms and to support patients in developing coping strategies to face financial, occupational, and relational challenges [30,31,32]. Coaching elements covered included social support engagement, stress and insomnia handling, meditation, and practical counseling related to returning to work and marital arrangement [32,33].

### 3.2. Meta-Analysis

**Figure 3:**
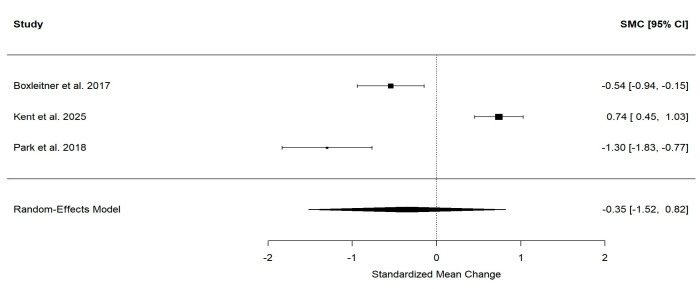
Forest plot for the pre–post change in stress levels following nurse coaching intervention [19,20,21].

A total of three studies were included in the analysis, comprising 112 participants. Heterogeneity among studies was very high and statistically significant (Q(2) = 55.31; *p* < 0.001; τ^2^ = 1.02; I^2^ = 96.2%, with a 95% CI: [85.89; 99.90]), thus a random-effects model (REML) was applied. The forest plot (Figure 3) indicated a small, non-significant reduction in stress levels following the nurse coaching interventions, with an overall SMCC of −0.35 (SE = 0.60; 95% CI: [−1.52, 0.82]; *p* = 0.556). Sensitivity analyses varying the within-subject correlation (r = 0.3, 0.5, 0.7) produced similar results, suggesting that the effect estimate was robust to this assumption (Supplementary File S2). Leave-one-out analyses showed that the pooled effect was highly influenced by individual studies: excluding Study 2 yielded a significant reduction in stress (SMCC = −0.90; 95% CI [−1.64, −0.16]; *p* = 0.017) and lower heterogeneity (I^2^ = 79.9%) (Supplementary File S3). These findings suggest a potential trend toward stress reduction after the intervention, and although the effect was not statistically significant, the results are highly sensitive to study-specific characteristics and should be interpreted with caution.

## 4. Discussion

Cancer very often represents a profound heavy psychological experience characterized by fear, anxiety, depression, uncertainty, and emotional distress. Beyond this critical psychological reaction, cancer-related treatment can considerably reduce patients’ quality of life (QoL), including after treatment completion. Emotional distress has also been associated with the activation of neuroendocrine stress pathways, increasing stress hormone secretion and immune dysregulation, which may further negatively influence health outcomes [1]. Within this scenario, factors which simultaneously address psychological, social, and practical stressors are essential to provide holistic oncology care. The findings highlighted in the present review suggest that nurse coaching represents a promising, though still evolving, approach to stress reduction among cancer patients.

Growing evidence indicates that nurse coaching can improve QoL and reduce stress by targeting multiple interrelated mechanisms. Central among these is the strengthening of social support [34,35,36]. Coaching-based interventions, including those adapted from the CARING model, emphasize the identification, mobilization, and optimization of both informal and formal support networks. Tools such as digital eco-mapping guide patients and caregivers in visualizing existing resources, recognizing unmet support needs, and identifying opportunities to engage family, friends, and community services. By reducing isolation and normalizing help-seeking behaviors, nurse coaching is a key contributor to alleviating cancer-related distress, particularly among underserved and rural populations, where access to support may be limited and reluctance to seek help is well-documented.

Another critical mechanism through which nurse coaching reduces stress is the enhancement of coping self-efficacy. Psychoeducational components improve understanding of the disease, expectations for treatment, symptom management, and recovery trajectories, thereby reducing uncertainty and fear [9]. Nurse coaching interventions frequently integrate practical skills such as stress management techniques, meditation, and insomnia management, equipping patients with tools to regulate emotional responses across the cancer trajectory [13]. By fostering a sense of competence and control, these interventions mitigate feelings of helplessness that commonly accompany cancer treatment.

Research indicates that when patients receive adequate support, clear information, and opportunities to participate in treatment decisions, they tend to report reduced anxiety, fewer symptom-related difficulties, and better compliance with their treatment plans [13]. Oncology nurses that use nurse coaching skills can help meet those needs by creating collaborative relationships with patients, acknowledging their emotional struggles, and adjusting care in response to changing circumstances [37]. Throughout the challenging and often uncertain course of cancer treatment, ongoing personalized interactions with a skilled Nurse Coach can help reduce the sense of being alone, overwhelmed, or powerless [2,38].

Nurse coaching contributes to stress reduction through patient-centered delivery and flexibility. Evidence from meditation-based interventions during radiation therapy demonstrates that self-guided, flexible formats can be as effective as coach-led approaches in reducing anxiety and distress. Patients receiving guided meditation have often cited scheduling constraints and treatment-related fatigue as barriers to participation, whereas self-directed options allowed for engagement at convenient times without added burden [30]. Nurse coaching models that offer flexible modalities—such as telephone-based sessions, digital platforms, or brief structured encounters—are particularly well suited to patients undergoing physically and emotionally demanding treatments and may promote sustained engagement over time [39,40].

Importantly, nurse coaching also addresses practical stressors, including barriers that frequently exacerbate psychological distress. Tailored guidance related to financial concerns, insurance navigation, return-to-work planning, and role transitions helps reduce external pressures that undermine emotional well-being. By integrating psychosocial and practical support, Nurse Coaching adopts a whole-person holistic approach to stress management rather than focusing solely on emotional symptoms [32]. This comprehensive scope aligns with patient-identified needs and reflects the realities of living with and beyond cancer.

The enCompass Carolina program provides a concrete example of how coaching-based interventions can be adapted to meet the needs of specific populations. Designed for caregivers of rural cancer patients, enCompass demonstrated feasibility and high acceptability, with caregiver participation (44%) and retention (75%) comparable to or exceeding those reported in similar interventions [20]. Adapted from the CARING model, the program emphasized caregiver-centered flexibility, support-seeking skills, and the use of a simple digital eco-mapping tool to identify and mobilize social support. The successful delivery of the intervention by trained lay health coaches underscores its scalability and potential for broader implementation, particularly in settings with limited healthcare resources. While preliminary improvements in caregiver outcomes were observed, further controlled studies are needed to establish efficacy and to clarify downstream effects on patient stress and healthcare utilization [20].

Despite the proliferation of interventions aimed at improving coping among cancer caregivers, relatively few have been successfully integrated into routine clinical practice [20]. Common barriers include insufficient funding and reimbursement mechanisms, limited provider awareness and training, and concerns regarding intervention fit across diverse care settings. These challenges were explicitly addressed in the design of enCompass through population-specific adaptation, in-clinic caregiver identification, tailoring to existing support networks, flexible delivery modalities, and a relatively low time burden. Such design features reflect core coaching principles and may enhance real-world feasibility and sustainability [41].

Similarly, individualized psychoeducational interventions for breast cancer patients illustrate how coaching-oriented strategies can improve emotional well-being and QoL [42]. By addressing patient-identified sources of distress across the cancer trajectory, these interventions enhanced disease understanding, prepared patients for treatment-related symptoms, and supported coping with financial, occupational, and relational challenges [43]. Coaching components—including facilitation of social support, stress management techniques, meditation, insomnia management, and counseling related to returning to work and marital adjustment—were associated with mild improvements in emotional outcomes, consistent with prior evidence supporting psychoeducation as an effective psychosocial intervention [44].

Despite these encouraging qualitative and mechanistic findings, the quantitative synthesis of available studies in this analysis revealed only a small, non-significant reduction in stress following nurse coaching interventions. Across three studies, comprising 112 participants, heterogeneity was very high, necessitating a random-effects model. The pooled standardized mean difference suggested a trend toward reduced stress (SMCC = −0.35, SE = 0.60; 95% CI: [−1.52, 0.82]; *p* = 0.556), but the effect did not reach statistical significance. However, although the results should be interpreted with caution due to the high heterogeneity observed (I^2^ = 96.2%) and limited number of studies included, this result probably reflects substantial variability in intervention content, delivery formats, populations, and outcome measurement, as well as limited sample sizes and methodological constraints. These factors highlight the need for cautious interpretation and underscore the importance of more rigorous, adequately powered studies.

Nurse coaching reduces stress among cancer patients and caregivers through multiple complementary mechanisms, including enhanced social support, strengthened coping skills and self-efficacy, flexible, patient-centered delivery, and attention to both emotional and practical stressors. While current quantitative evidence suggests only modest, non-significant effects on stress, the consistency of the theoretical rationale and the qualitative benefits support continued investigation. Future research should prioritize pragmatic, well-controlled trials to evaluate effectiveness, implementation, and sustainability, thereby clarifying the role of nurse coaching as an integral component of routine oncology care [45].

However, it should be considered that our findings were not statistically significant, and the number of studies included was very small. Thus, the results should be interpreted with caution, since the pooled effect estimate might be unstable, and heterogeneity appeared to be difficult to assess and could not be meaningfully explored. Additionally, publication bias assessment tools, such as funnel plots, were not reliable with so few studies. Finally, several coaching interventions have been included as a coaching categorization without any differentiation among differences in interventions.

## 5. Conclusions

This review highlights nurse coaching as a holistic and patient-centered approach with the potential to mitigate stress and enhance quality of life among individuals affected by cancer. By addressing the multifaceted nature of cancer-related distress, nurse coaching targets not only emotional responses but also social and practical stressors that contribute to sustained psychological burden. Through mechanisms such as strengthening social support networks, enhancing coping self-efficacy, providing tailored psychoeducation, and offering flexible, accessible modes of delivery, coaching-based interventions align closely with the complex needs of patients and caregivers across the cancer trajectory.

Although the quantitative evidence synthesized in this analysis demonstrated only a small and non-significant reduction in stress, the observed trends, combined with consistent qualitative and mechanistic findings, suggest meaningful potential benefits. High heterogeneity and methodological limitations across studies underscore the need for cautious interpretation and highlight gaps in the current evidence base rather than a lack of therapeutic promise. Programs such as enCompass Carolina [20] further demonstrate the feasibility, acceptability, and scalability of coaching models, particularly in underserved and rural settings where psychosocial support is often limited.

Future research should focus on rigorously designed, adequately powered, and pragmatically oriented trials to better evaluate the effectiveness and sustainability of nurse coaching interventions. Attention to standardization of core coaching components while preserving flexibility and personalization will be critical to advancing implementation in routine oncology care. Overall, nurse coaching skills represent a promising adjunct to comprehensive cancer care, with the potential to support psychological well-being, empower patients and caregivers, and address the broader determinants of stress throughout the cancer experience.

## Figures and Tables

**Table 1:** The PIO tool for the present systematic review and meta-analysis.

Population	Subjects Experiencing or Caring for Cancer
Intervention	Coaching intervention
Outcome	Stress level assessments

**Table 2:** Nurse coaching interventions in oncology care to reduce stress in the screened studies (n = 3).

Author(s) Publication Years	Study Design EBN Quality Assessment	Participants No.	Age	Cancer Typology	Coaching Intervention			
					Intervention	Setting	Intent	Description
Boxleitner et al. 2017 [19]	Randomized clinical trial Level I	n = 29 caregivers	59.1 ± 10.6 years	Head and neck cancer	Meditation with a coach	Patients experienced a 20 min/week coach-guided meditation session immediately after a radiation treatment. Sessions were organized on the same day and at the same time every week attended by the same nurse coach.	To improve mitigation and calm to manage anxiety and depression, encouraging present-moment awareness to reduce stress.	Structured meditation approaches were presented in each coached session. Each session started with a short conversation to provide comfort, followed by guided mindful breathing and body-scanning exercises. Participants were invited to use these approaches during moments of tension or anxiety.
Kent et al. 2025 [20]	Single-arm, single-site study Level III	n = 51 patients; n = 59 caregivers	Patients: 65.7 ± 13.8; Caregivers: 59.7 ± 12.5	Breast, prostate, digestive (colon, small intestine, other digestive, anal, rectal), leukemia, lymphoma, multiple myeloma	8-week supportive care intervention	A web-based support mapping tool (eSNAP encompass) and personalized sessions with an interventionist and a workbook.	To recognize and organize informal and formal support from friends and family in the cancer center.	The eSNAP tool was structured in six dimensions of social support: in-home, informational, communication and coordination, financial, emotional and spiritual and transportation and delivery.
Park et al. 2018 [21]	Quasi-experimental trial Level IV	n = 25 patients experimental group; n = 22 patients control group.	40–50 years	Breast cancer	Distress management—integrated psychoeducational program	Immediately after diagnosis, pre-surgery, before starting chemotherapy and after completing primary treatment, the experimental group received three sessions of individual education using educational booklets.	To assess the effects of an integrated psychoeducational program for distress management.	Each session, participants first received 5–10 min to recognize any newly emerging issues, additional questions, or requests for information following the initial education. Re-education was provided, like weight management, dietary therapy for breast cancer patients, scalp care, laughter therapy, meditation, and insomnia management.

## Data Availability

The original contributions presented in this study are included in the article/Supplementary Material. Further inquiries can be directed to the corresponding author.

## Supplementary Materials

The following supporting information can be downloaded at: https://www.mdpi.com/article/10.3390/healthcare14070840/s1, Supplementary File S1. Search strings carried out to perform this systematic and meta-analysis study. Supplementary File S2. SMCC sensitivity analysis across assumed within-subject correlations. Supplementary File S3. Leave-One-Out Sensitivity Analysis.

## References

[1] Vitale E., Motamed-Jahromi M., Parvaresh-Masoud M., Lagattolla F., Cormio C., Romito F., Massafra R. (2025). Nurse coaching can improve the quality of life and immune-endocrine condition in hospitalized cancer patients. Endocr. Metab. Immune Disord. Drug Targets.

[2] Vignjević Petrinović S., Milošević M.S., Marković D., Momčilović S. (2023). Interplay between stress and cancer—A focus on inflammation. Front. Physiol..

[3] Wu Y., Zhou L., Zhang X., Yang X., Niedermann G., Xue J. (2022). Psychological distress and eustress in cancer and cancer treatment: Advances and perspectives. Sci. Adv..

[4] Vitale E., Maistrello L., Motamed-Jahromi M., Avino K., Rizzo A. (2024). Effect of Health Coaching on Anxiety and Depression Conditions among Cancer Patients: A Systematic Review and Meta-Analysis of Randomized Controlled Trials. J. Psychopathol..

[5] Vitale E., Avino K., Mea R., Comes M.C., Bove S., Conte L., Lupo R., Rubbi I., Carvello M., Botti S. (2024). Variations in the Five Facets of Mindfulness in Italian Oncology Nurses according to Sex, Work Experience in Oncology, and Shift Work. Healthcare.

[6] Bradley J., Moore L.W. (2020). Best practices for working with a professional coach. Nurs. Manag..

[7] Jordan K.J., Tsai P.F., Heo S., Bai S., Dailey D., Beck C.K., Butler L.M., Greenwood R.L. (2017). Pilot testing a coaching intervention to improve certified nursing assistants’ dressing of nursing home residents. Res. Gerontol. Nurs..

[8] Westcott L. (2016). How coaching can play a key role in the development of nurse managers. J. Clin. Nurs..

[9] Dermody E. (2021). Nurse coaching: Providing holistic care to patients with cancer. Clin. J. Oncol. Nurs..

[10] Walsh S., Simmons Z., Miyamoto S., Geronimo A. (2025). A Nurse Coaching Intervention to Improve Support to Individuals Living with ALS. Amyotroph. Lateral Scler. Front. Degener..

[11] Barr J.A., Tsai L.P. (2021). Health Coaching Provided by Registered Nurses Described: A Systematic Review and Narrative Synthesis. BMC Nurs..

[12] Bian J., Chen F., Fang S., Wang Y. (2025). Self-Compassion Intervention Programs for Nurses: A Scoping Review. Healthcare.

[13] Edgman-Levitan S., Schoenbaum S.C. (2021). Patient-Centered Care: Achieving Higher Quality by Designing Care through the Patient’s Eyes. Isr. J. Health Policy Res..

[14] Almutairi M., Almutairi A.A., Alodhialah A.M. (2025). A cross-sectional study on the impact of nurse-led health coaching on oncology patient outcomes. J. Multidiscip. Healthc..

[15] Bailey A.K., Tao H., Sawyer A.T. (2025). From Research to Practice: Implementing an Evidence-Based Intervention for Nurse Well-Being in a Healthcare System. Healthcare.

[16] Page M.J., McKenzie J.E., Bossuyt P.M., Boutron I., Hoffmann T.C., Mulrow C.D., Shamseer L., Tetzlaff J.M., Akl E.A., Brennan S.E. (2021). The PRISMA 2020 Statement: An Updated Guideline for Reporting Systematic Reviews. BMJ.

[17] Donovan K.A., Grassi L., McGinty H.L., Jacobsen P.B. (2014). Validation of the Distress Thermometer worldwide: State of the science. Psycho-Oncology.

[18] Sterne J.A.C., Savović J., Page M.J., Elbers R.G., Blencowe N.S., Boutron I., Cates C.J., Cheng H.Y., Corbett M.S., Eldridge S.M. (2019). RoB 2: A revised tool for assessing risk of bias in randomised trials. BMJ.

[19] Boxleitner G., Jolie S., Shaffer D., Pasacreta N., Bai M., McCorkle R. (2017). Comparison of Two Types of Meditation on Patients’ Psychosocial Responses During Radiation Therapy for Head and Neck Cancer. J. Altern. Complement. Med..

[20] Kent E.E., Deal A.M., Sperry S.S., Nakamura Z.M., Gellin M., Kovacs J., Martin C., Reblin M. (2025). Feasibility and Acceptability of an 8-Week Supportive Care Intervention for Cancer Caregivers Adapted for Rural Settings. Psycho-Oncology.

[21] Park J.H., Chun M., Jung Y.S., Bae S.H., Jung Y.M. (2018). Psychoeducational Approach to Distress Management of Newly Diagnosed Patients with Breast Cancer. J. Korean Acad. Nurs..

[22] Melnyk B.M., Fineout-Overholt E. (2022). Evidence-Based Practice in Nursing & Healthcare: A Guide to Best Practice.

[23] Morris S.B., DeShon R.P. (2002). Combining effect size estimates in meta-analysis with repeated measures and independent-groups designs. Psychol. Methods.

[24] Borenstein M., Hedges L.V., Higgins J.P.T., Rothstein H.R. (2009). Introduction to Meta-Analysis.

[25] Higgins J.P.T., Thomas J., Chandler J., Cumpston M., Li T., Page M.J., Welch V.A. (2023). Cochrane Handbook for Systematic Reviews of Interventions.

[26] Deeks J.J., Higgins J.P., Altman D.G., Higgins J.P.T., Thomas J., Chandler J., Cumpston M., Li T., Page M.J., Welch V.A. (2019). Analysing data and undertaking meta-analyses. Cochrane Handbook for Systematic Reviews of Interventions.

[27] Higgins J.P., Green S. (2008). Cochrane Handbook for Systematic Reviews of Interventions.

[28] Lewis S., Clarke M. (2001). Forest plots: Trying to see the wood and the trees. BMJ.

[29] Chen A.M., Daly M.E., Farwell D.G., Vazquez E., Courquin J., Lau D.H., Purdy J.A. (2014). Quality of Life among Long-Term Survivors of Head and Neck Cancer Treated by Intensity Modulated Radiotherapy. JAMA Otolaryngol.–Head Neck Surg..

[30] Badr H., Gupta V., Sikora A., Posner M. (2014). Psychological Distress in Patients and Caregivers over the Course of Radiotherapy for Head and Neck Cancer. Oral Oncol..

[31] Kent E.E., Tan K.R., Nakamura Z.M., Kovacs J., Gellin M., Deal A., Park E.M., Reblin M. (2024). Building On and Tailoring To: Adapting a Cancer Caregiver Psychoeducational Intervention for Rural Settings. Cancer Med..

[32] Reblin M., D’Almeida H., Barrios-Monroy V., McCormick R., Rodriguez L., Walters K., Sutton S.K., Zebrack B., Forsyth P., Byrne M.M. (2022). Training Cancer Caregiver Navigators: Experiences from Implementing the eSNAP and Caregiver Navigator Intervention. Support. Care Cancer.

[33] Holland J.C., Andersen B., Breitbart W.S., Buchmann L.O., Compas B., Deshields T.L., Dudley M.M., Fleishman S., Fulcher C.D., Greenberg D.B. (2013). Distress Management. J. Natl. Compr. Cancer Netw..

[34] Park J.H., Chun M., Jung Y.S., Bae S.H. (2017). Predictors of Psychological Distress Trajectories in the First Year after a Breast Cancer Diagnosis. Asian Nurs. Res..

[35] Ai Z.P., Gao X.L., Li J.F., Zhou J.R., Wu Y.F. (2017). Changing Trends and Influencing Factors of the Quality of Life of Chemotherapy Patients with Breast Cancer. Chin. Nurs. Res..

[36] Matsuda A., Yamaoka K., Tango T., Matsuda T., Nishimoto H. (2014). Effectiveness of Psychoeducational Support on Quality of Life in Early-Stage Breast Cancer Patients: A Systematic Review and Meta-Analysis of Randomized Controlled Trials. Qual. Life Res..

[37] Richardson C., Wicking K., Biedermann N., Langtree T. (2023). Coaching in Nursing: An Integrative Literature Review. Nurs. Open.

[38] Cummings G.G., Hewko S.J., Wang M., Wong C.A., Laschinger H.K.S., Estabrooks C.A. (2018). Impact of Managers’ Coaching Conversations on Staff Knowledge Use and Performance in Long-Term Care Settings. Worldviews Evid. Based Nurs..

[39] Premanandan S., Ahmad A., Cajander Å., Ågerfalk P., van Gemert-Pijnen L. (2023). Design Suggestions for a Persuasive E-Coaching Application: A Study on Informal Caregivers’ Needs. Digit. Health.

[40] Tolotti A., Barello S., Vignaduzzo C., Liptrott S.J., Valcarenghi D., Nania T., Sari D., Bonetti L. (2022). Patient engagement in oncology practice: A qualitative study on patients’ and nurses’ perspectives. Int. J. Environ. Res. Public Health.

[41] Shaban M.M., Sharaa H.M., Amer F.G.M., Shaban M. (2024). Effect of Digital-Based Nursing Intervention on Knowledge of Self-Care Behaviors and Self-Efficacy of Adult Clients with Diabetes. BMC Nurs..

[42] Alanazi A., Mitchell G., Al Halaiqa F.N., Khraim F., Craig S. (2026). Digital Interventions for Palliative Care Education for Nursing Students: A Systematic Review. Nurs. Rep..

[43] Flaubert J.L., Le Menestrel S., Williams D.R., Wakefield M.K., National Academies of Sciences, Engineering, and Medicine, National Academy of Medicine, Committee on the Future of Nursing 2020–2030 (2021). Chapter 10, Supporting the Health and Professional Well-Being of Nurses. The Future of Nursing 2020–2030: Charting a Path to Achieve Health Equity.

[44] Charles-Rodriguez U., Ngwezi D.P., Damag S., Johnson N., Bharwani A., Ladha T., Salami O. (2025). Uncovering Systemic Barriers Related to Equity, Diversity and Inclusion in Child Health Research: A Scoping Review Addressing Marginalised Communities. BMJ Glob. Health.

[45] Galway K., Black A., Cantwell M., Cardwell C.R., Mills M., Donnelly M. (2012). Psychosocial Interventions to Improve Quality of Life and Emotional Wellbeing for Recently Diagnosed Cancer Patients. Cochrane Database Syst. Rev..

