# Supplementary material for: Nurse Coaching in Oncology Care to Reduce Stress: A Systematic Review and Meta-Analysis
Source: Healthcare (Basel). 2026 Mar 25;14(7):840. doi: 10.3390/healthcare14070840 (PMC13073765; doi:10.3390/healthcare14070840)
Supplement: Supplementary file 1 [file healthcare-14-00840-s001.zip › healthcare-4170398-supplementary.pdf]

**Supplementary File 1. Search strings carried out to perform this systematic and meta-analysis study.**

| Database | Results |
|---|---|
| **CINAHL**<br>**coaching AND intervention AND oncology nursing AND stress** | 2 |
| **Embase**<br>('coaching'/exp OR 'coaching') AND ('intervention'/exp OR 'intervention') AND ('oncology nursing'/exp OR 'oncology nursing') AND ('stress'/exp OR 'stress') | 5 |
| **PubMed**<br>**(((Coaching) AND (Intervention)) AND (Oncology Nursing)) AND (Stress)**<br>("coach"[All Fields] OR "coach s"[All Fields] OR "coached"[All Fields] OR "coaches"[All Fields] OR "mentoring"[MeSH Terms] OR "mentoring"[All Fields] OR "coaching"[All Fields]) AND ("intervention s"[All Fields] OR "interventions"[All Fields] OR "interventive"[All Fields] OR "methods"[MeSH Terms] OR "methods"[All Fields] OR "intervention"[All Fields] OR "interventional"[All Fields]) AND ("oncology nursing"[MeSH Terms] OR ("oncology"[All Fields] AND "nursing"[All Fields]) OR "oncology nursing"[All Fields]) AND ("stress"[All Fields] OR "stressed"[All Fields] OR "stresses"[All Fields] OR "stressful"[All Fields] OR "stressfulness"[All Fields] OR "stressing"[All Fields])<br>**Translations**<br>**Coaching:** "coach"[All Fields] OR "coach's"[All Fields] OR "coached"[All Fields] OR "coaches"[All Fields] OR "mentoring"[MeSH Terms] OR "mentoring"[All Fields] OR "coaching"[All Fields]<br>**Intervention:** "intervention's"[All Fields] OR "interventions"[All Fields] OR "interventive"[All Fields] OR "methods"[MeSH Terms] OR "methods"[All Fields] OR "intervention"[All Fields] OR "interventional"[All Fields]<br>**Oncology Nursing:** "oncology nursing"[MeSH Terms] OR ("oncology"[All Fields] AND "nursing"[All Fields]) OR "oncology nursing"[All Fields]<br>**Stress:** "stress"[All Fields] OR "stressed"[All Fields] OR "stresses"[All Fields] OR "stressful"[All Fields] OR "stressfulness"[All Fields] OR "stressing"[All Fields] | 27 |
| **Scopus**<br>('coaching'/exp OR 'coaching') AND ('intervention'/exp OR 'intervention') AND ('oncology nursing'/exp OR 'oncology nursing') AND ('stress'/exp OR 'stress') | 3 |
| **WoS**<br>**coaching** (Topic) and **intervention** (All Fields) and **oncology nursing** (All Fields) and **stress** (All Fields) | 15 |

**Supplementary File 2.** SMCC sensitivity analysis across assumed within-subject correlations.

| Assumed r | SMCC | SE | 95% CI | P-value | $\tau^2$ | $I^2$ (%) | Q (df=2) | P-value(Q) |
|---|---|---|---|---|---|---|---|---|
| 0.3 | -0.31 | 0.52 | [-1.33; 0.70] | 0.547 | 0.77 | 95.3 | 44.60 | < 0.001 |
| 0.5 | -0.35 | 0.60 | [-1.52; 0.82] | 0.556 | 1.02 | 96.2 | 55.31 | < 0.001 |
| 0.7 | -0.40 | 0.73 | [-1.83; 1.03] | 0.579 | 1.54 | 97.2 | 74.26 | < 0.001 |

SMCC = Standardized Mean Change with Control (pre-post design), calculated assuming the within-subject correlation (r) indicated in the first column; SE = standard error of the effect; 95% CI = 95% confidence interval for SMCC; P-value = significance of the overall effect; $\tau^2$ = between-study variance; $I^2$ = proportion of total variability due to heterogeneity, Q = Cochran's Q statistic, P-value(Q) = significance of the heterogeneity test.

**Supplementary File 3.** Leave-One-Out Sensitivity Analysis

| Study removed | SMCC | SE | P-value | 95% CI | $\tau^2$ | $I^2$ (%) | $H^2$ |
|---|---|---|---|---|---|---|---|
| Boxleitner et al. 2017 | -0.27 | 1.02 | 0.790 | [-2.26; 1.73] | 2.03 | 97.7 | 43.6 |
| Kent et al. 2025 | -0.90 | 0.38 | **0.017** | [-1.64; -0.16] | 0.23 | 79.9 | 5 |
| Park et al. 2018 | 0.11 | 0.64 | 0.870 | [-1.15; 1.36] | 0.79 | 96.2 | 26.3 |

SMCC = Standardized Mean Change with Control (pre-post design); $\tau^2$ = between-study variance; $I^2$ = proportion of total variability due to heterogeneity; $H^2$ = total variability / sampling variability; 95% CI = confidence interval.
